# New look at the 20 mmHg ICP threshold

**DOI:** 10.1186/cc13648

**Published:** 2014-03-17

**Authors:** FG Guiza, BD Depreitere, IP Piper, GV Van den Berghe, GM Meyfroidt

**Affiliations:** 1 University Hospitals Leuven, Belgium; 2 Southern General Hospital, Glasgow, UK

## Introduction

We present a method based on minute-by-minute ICP monitoring and outcome (GOS) to visualize in a clinically useful way the dynamic aspects of secondary injury.

## Methods

A retrospective analysis of data from 165 adult patients from the Brain-IT database [1]. A color-coded contour plot was made for the association between good outcome and the number of secondary insults of ICP during the ICU stay, as defined by continuous values of insult duration and thresholds.

## Results

**Figure 1:**
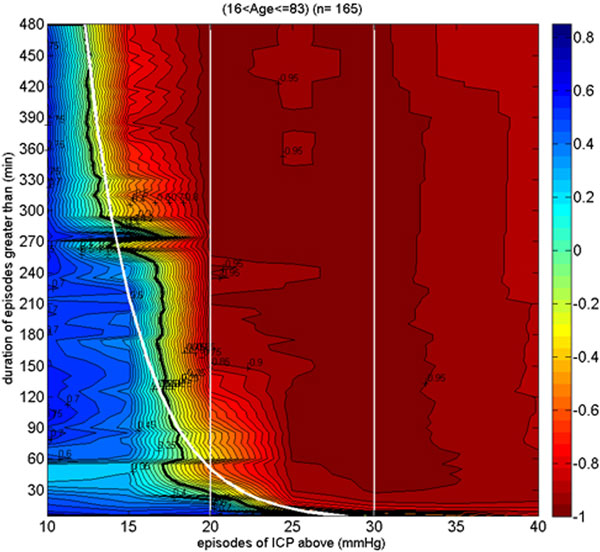


Figure 1 visualizes in blue the region associated with good outcome for continuous values of thresholds and durations of secondary insults. The thick black line indicates the transition towards the negative association region in red. A best-fit exponential curve is superimposed. This clearly shows that insults below 20 mmHg of ICP can be detrimental if sustained for long periods. Alternatively, they can be tolerated for higher thresholds but only for shorter periods.

## Conclusion

Secondary injury is dynamic such that not only thresholds but also insult duration are relevant. The proposed visualization goes beyond the static 20 mmHg ICP threshold introduced in [2] and provides a more accurate representation that could help the clinician in identifying when the patient's outcome could be making a turn for the worse.
